# Hepatocellular Carcinoma With Cardiac Metastasis Presenting as Presyncope

**DOI:** 10.7759/cureus.24926

**Published:** 2022-05-11

**Authors:** Muhammad S Haq, Tehreem Fatima, John Caplan

**Affiliations:** 1 Internal Medicine, St. Francis Medical Center, Trenton, USA; 2 Cardiology, Seton Hall University/St. Francis Medical Center, Trenton, USA

**Keywords:** ascitis, right heart failure, transarterial chemoembolization (tace), right atrial thrombus, hepatocellular carcinoma (hcc)

## Abstract

Hepatocellular carcinoma (HCC) is a common primary tumor of the liver that is highly invasive and can even invade the portal and hepatic veins in later stages. In this report, we present one such rare case of HCC invading the right atrium. A 69-year-old male patient recently diagnosed with HCC secondary to hepatitis C presented to the hospital after experiencing an episode of near syncope. On examination, he had a distended abdomen consistent with ascites with positive fluid shift, elevated jugular vein distention (JVD), and bilateral pitting lower extremity edema. He had an elevated alkaline phosphatase of 298 U/L (34-104) with a total bilirubin of 2.7mg/dL (0.3-1) and a D-dimer of 1.67ugFEU/mL (<0.5). On admission, CT scan of the chest and abdomen showed extensive invasion of the liver by neoplasm and a large 7 cm mass extending from the intrahepatic inferior vena cava to the right atrium. A transthoracic echocardiogram confirmed this, which also better visualized the cardiac anatomy. Due to the extent of the disease, the patient ultimately opted for palliative care. The prognosis for patients with HCC who have an invasion of the right atrium remains dismal, with a median survival of only five months. Surgical extraction of the thrombus with resection of the tumor, liver transplantation, and systemic chemotherapy are some of the treatment modalities employed in such patients; however, historically, the median survival has remained only a few months. With the advent of new techniques and a better understanding of the disease, this seems to be changing and a curative approach can now be considered.

## Introduction

Primary cardiac tumors are extremely rare with some estimates reporting incidence rates from 0.001% to 0.28%. On the other hand, metastatic cancers to the heart are relatively more common with pleural mesothelioma, melanoma, and lung carcinomas being the most frequently encountered [1]. Hepatocellular carcinoma (HCC) is the most common primary tumor of the liver, representing 90% of all liver cancers, and is the second leading cause of cancer-related deaths in the world [2]. It is also highly invasive with widespread metastatic sites including lungs, brain, bones, and adrenals [3]. In advanced stages, it can also invade the hepatic and portal veins and can metastasize directly to the right atrium of the heart. This is extremely rare and some studies suggest that the incidence of direct invasion of the heart could be as low as 1-4% [3-6]. This article reports one such rare case of HCC invading the right atrium.

## Case presentation

A 69-year-old male recently diagnosed with HCC secondary to hepatitis C presented to the hospital after experiencing an episode of near syncope. He described his episode as lightheadedness and weakness that started as he was ambulating and subsided within two minutes after he sat down. He also endorsed reduced oral intake secondary to abdominal pain. On examination, he had a distended abdomen consistent with ascites with positive fluid shift, elevated jugular vein distention (JVD), and bilateral pitting lower extremity edema. EKG on arrival revealed sinus tachycardia, left atrial enlargement, and left anterior fascicular block. He had recently learned of his diagnosis of HCC approximately four months ago but had chosen not to pursue chemotherapy. He had elevated liver enzymes, including aspartate aminotransferase (AST), alanine aminotransferase (ALT), alkaline phosphatase (ALP), and bilirubin on admission. He also had a deranged coagulation profile with mildly increased INR, PT, and PTT. His albumin at the time of admission was in the normal range (Table 1).

**Table 1:** Abnormal laboratory investigations at the time of admission with reference ranges.

	n/unit	Reference range
Alkaline phosphatase (ALP)	298 U/L	34-104 U/L
Total Bilirubin	2.7 mg/dL	0.3-1 mg/dL
Aspartate Aminotransferase (AST)	104 U/L	13-39 U/L
Alanine Aminotransferase (ALT)	57 U/L	7-52 U/L
D-dimer	1.67 ugFEU/mL	<0.5
Prothrombin time (PT)	16.4 secs	12.2-14.8 secs
Partial thromboplastin time (PTT)	39.5 secs	24.1-36.3 secs
International Randomized Ratio (INR)	1.3	0.8-1.1
Albumin	3.5 g/dL	3.5-5 g/dL

**Figure 1:**
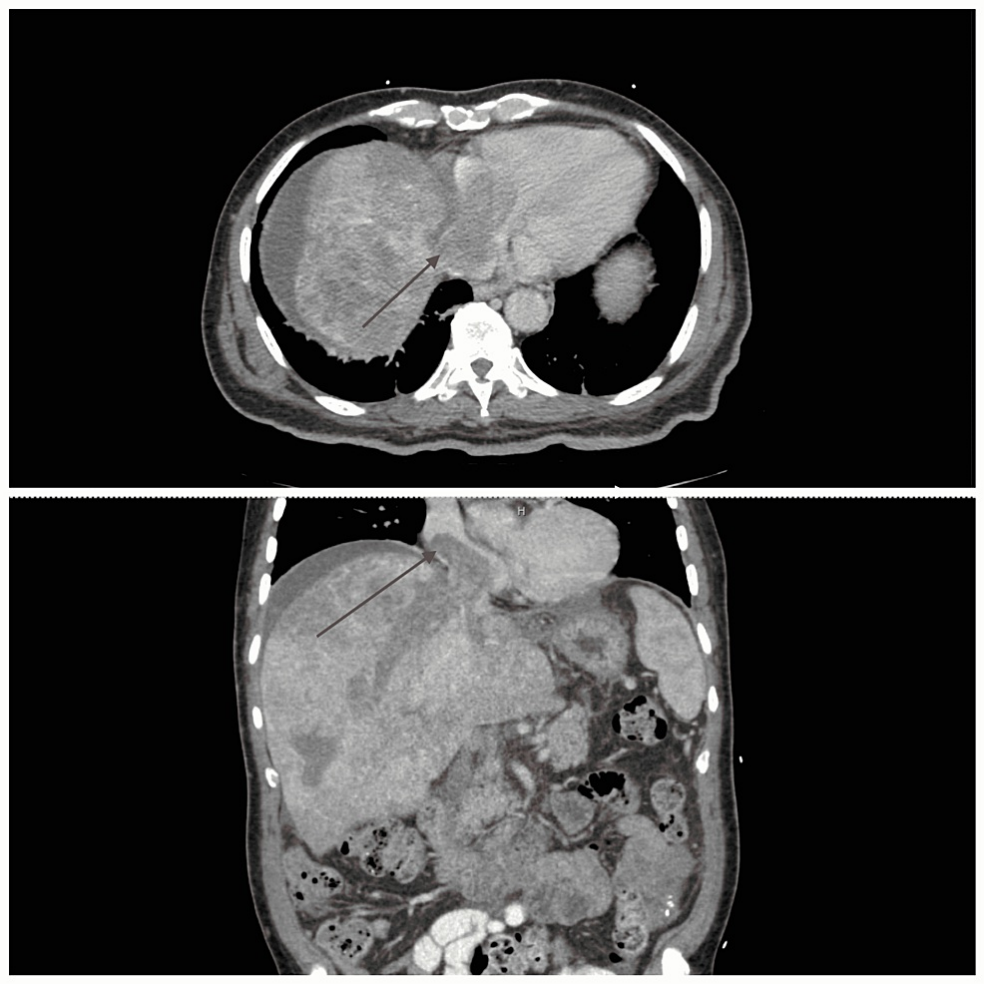
CT scan of the abdomen in the axial plane (top) and coronal plane (bottom) showing replacement of the liver with tumor. A 7cm tumor thrombus extending well into the right atrium with portal vein thrombosis (red arrow).

On admission, a CT scan of the chest and abdomen showed extensive invasion of the liver by neoplasm and a large 7cm mass extending from the intrahepatic inferior vena cava (IVC) to the right atrium (Figure 1). 

A transthoracic echocardiogram was done for better evaluation of the findings. It was suggestive of a large echogenic mass within the right atrium measuring 6 x 3.5cm (Video 1). A multidisciplinary discussion was held with the patient and treatment options were presented to him, but he ultimately opted for hospice care and was discharged to home hospice care. 

**Video 1:** Apical four-chamber view of the heart showing thrombus extending from the IVC into the right atrium. IVC: inferior vena cava

## Discussion

HCC usually invades the IVC and the right atrium by extending through the hepatic veins [4]. The prognosis for patients with HCC who have an invasion of the right atrium remains dismal, with a median survival of only five months with symptomatic management [4]. This condition is associated with several cardiopulmonary complications including pulmonary embolism, heart failure, lethal arrhythmias, and systemic metastasis [5]. The most common causes of death are heart failure and sudden cardiac death, reported in as much as 25% of the patients [5]. 

The clinical picture of HCC with right atrial extension is varied and depends mainly on the tumor size. A study done by Liu et al. reported some of the most common ways this disease process can present. It concluded that most patients remained asymptomatic at the time of diagnosis (39.5%), 37.5% of the patients had bilateral lower extremity edema, and 31.3% had exertional dyspnea [7]. Another study documented that at least 10% of patients with HCC, with or without cardiac metastasis, will have ascites [1]. Other clinical manifestations include syncope/presyncope, chest pain, cough, and hemoptysis [5]. These symptoms are manifestations of primary liver dysfunction as well as cardiac metastasis leading to right-sided heart failure. 

Once HCC has invaded the right atrium, there are no official guidelines on the management of the disease [5]. It is generally accepted that patients with HCC with extension into the right atrium are poor surgical candidates due to the complexities involved in the resection of the tumor in the IVC and the right atrium [8,9]. Other limiting factors of the surgical intervention include poor liver reserve, potential postoperative complications, and early recurrence [10]. Non-surgical approaches like the transarterial chemoembolization (TACE) procedure, chemotherapy, and radiotherapies have been attempted to palliate and improve quality of life. The results with these modalities have been poor with some studies reporting a median survival rate with the TACE procedure at 9.2 months, and systemic chemotherapy with sorafenib at 10.7 months [8]. A fascinating recent case report was published by Qiu et al., which describes a case of metastatic HCC with thrombi extending to IVC and right atrium, treated with hepatectomy, resection of the involved IVC, and right atrial thrombus, and reconstruction of the resected IVC [9]. The authors report that the patient remained disease-free at a 10-month follow-up [9]. Another case was reported by Li et al. who described the use of percutaneous microwave ablations with the help of TACE to label the tumor margins. As per the authors, they were able to completely ablate the tumor and the patient remained disease-free at a sixteen-month follow-up [4]. Unfortunately, we could not attempt any of these therapies with our patient due to the extent of the disease and the patient preference for palliation.

## Conclusions

As discussed, HCC with IVC and right atrium invasion has a poor prognosis. Although multiple therapies have been discussed, none have been studied extensively due to the rarity of this condition. Early detection and initiation of treatment in the earlier stages is the best course of action for HCC. Historically, when the right atrium of the heart is involved, management has been mostly palliative, but with the advent of new techniques and a better understanding of the disease, curative management can now be considered. 
